# A Case of Bilateral Pneumothorax With COVID-19 Infection

**DOI:** 10.7759/cureus.51081

**Published:** 2023-12-25

**Authors:** Andrew J Bergeron, Chinenye Emeshiobi, Nkolika Nwankwo, Mohankumar Doraiswamy

**Affiliations:** 1 Family Medicine, Mercy Hospital, Fort Smith, USA; 2 Internal Medicine, Mercy Hospital, Fort Smith, USA; 3 Internal Medicine, Nephrology, and Critical Care, Mercy Hospital, Fort Smith, USA

**Keywords:** respiratory failure, acute respiratory distress syndrome, spontaneous pneumothorax, bilateral pneumothorax, covid-19

## Abstract

Bilateral spontaneous pneumothorax is a serious complication of coronavirus disease 2019 (COVID-19). The incidence of any spontaneous pneumothorax in patients with the aforementioned viral infection when hospitalized is about 1%. Treatment can involve management such as oxygen support, tube thoracostomy, pleurodesis, or even invasive surgery. The associated mortality with this complication is about 33% to 52%. We present a case of bilateral pneumothorax in a patient diagnosed with COVID-19 without any history of smoking or underlying lung disease. Careful vigilance and close monitoring of this serious complication are mandatory in inpatients.

## Introduction

COVID-19 is a disease caused by the novel coronavirus SARS-CoV-2 (severe acute respiratory syndrome coronavirus 2), discovered in 2019. It is a viral respiratory illness that can potentially lead to multiple pulmonary and extrapulmonary complications, such as acute respiratory distress syndrome (ARDS), pneumonia, myocarditis, heart failure, acute liver injury, acute kidney injury, and hypercoagulability [1-3]. The spectrum of symptoms ranges from upper respiratory issues like cough and congestion to more severe conditions such as ARDS [3]. Spontaneous pneumothorax is a known respiratory complication associated with COVID-19, with an incidence of about 1% in hospitalized patients and a reported mortality rate ranging from 33% to 52% [4-7]. However, the occurrence of bilateral pneumothorax is exceedingly rare. In light of this, we report a case involving a 79-year-old woman who presented with acute respiratory failure secondary to COVID-19, which was further complicated by bilateral pneumothoraces.

## Case presentation

**Figure 1:**
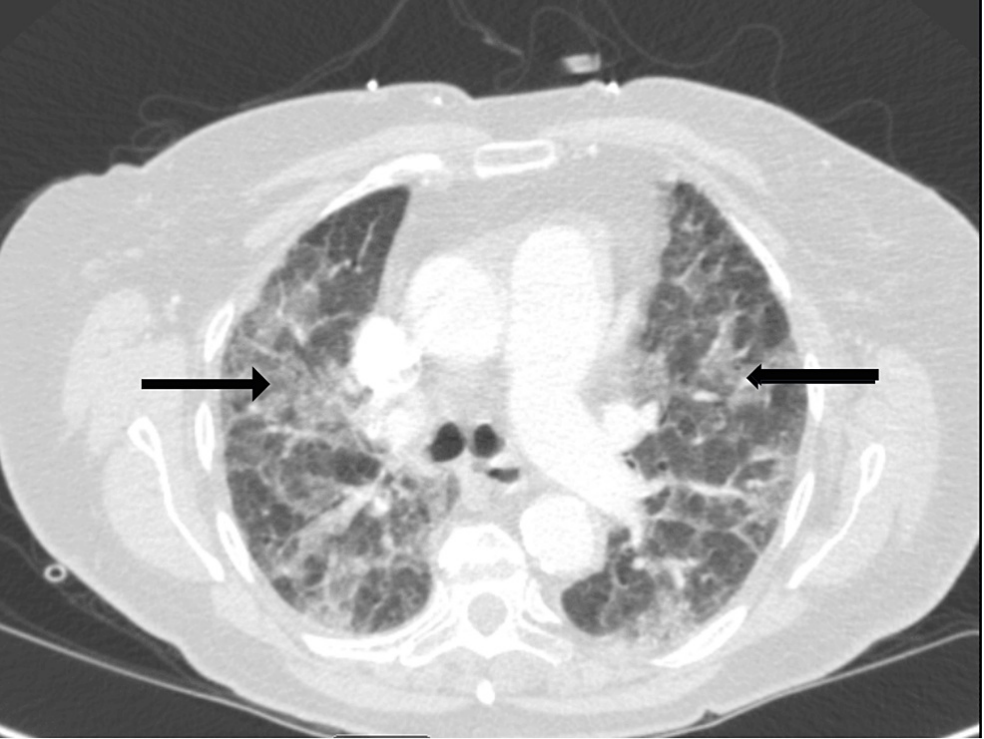
CT angiography of the chest showing scattered patchy ground glass opacities in bilateral lung fields.

A 79-year-old Caucasian, non-smoking female with a past medical history of hypertension, diastolic heart failure, type 2 diabetes mellitus, hyperlipidemia, and stage four chronic kidney disease was transferred to the intensive care unit (ICU) from a long-term acute care setting with worsening hypoxic respiratory failure. She had a prolonged and complicated stay at an outside hospital for five days, where she was treated for atypical pneumonia, urinary tract infection, and COVID-19 before being transferred to our ICU. Upon presentation to the outside facility, she exhibited no significant leukocytosis but did show some elevation in nonspecific anti-inflammatory markers such as C-reactive protein and ferritin, at 41 mg/L and 207 ng/mL, respectively. Her initial COVID-19 testing was negative. Due to acute dyspnea and an elevated D-dimer (1.34 ng/ml), she underwent computed tomography angiography of the chest, which was negative for pulmonary embolus but revealed scattered patchy ground-glass opacities suggestive of atypical pneumonia (Figure 1). An echocardiogram showed a normal ejection fraction with normal valvular function.

Also, her urine culture was positive for *Klebsiella pneumoniae *and *Escherichia coli*. She was treated with azithromycin, ceftriaxone, corticosteroids, and intravenous fluids. Owing to her worsening acute hypoxic respiratory failure, she was transferred to our hospital for further management.

**Figure 2:**
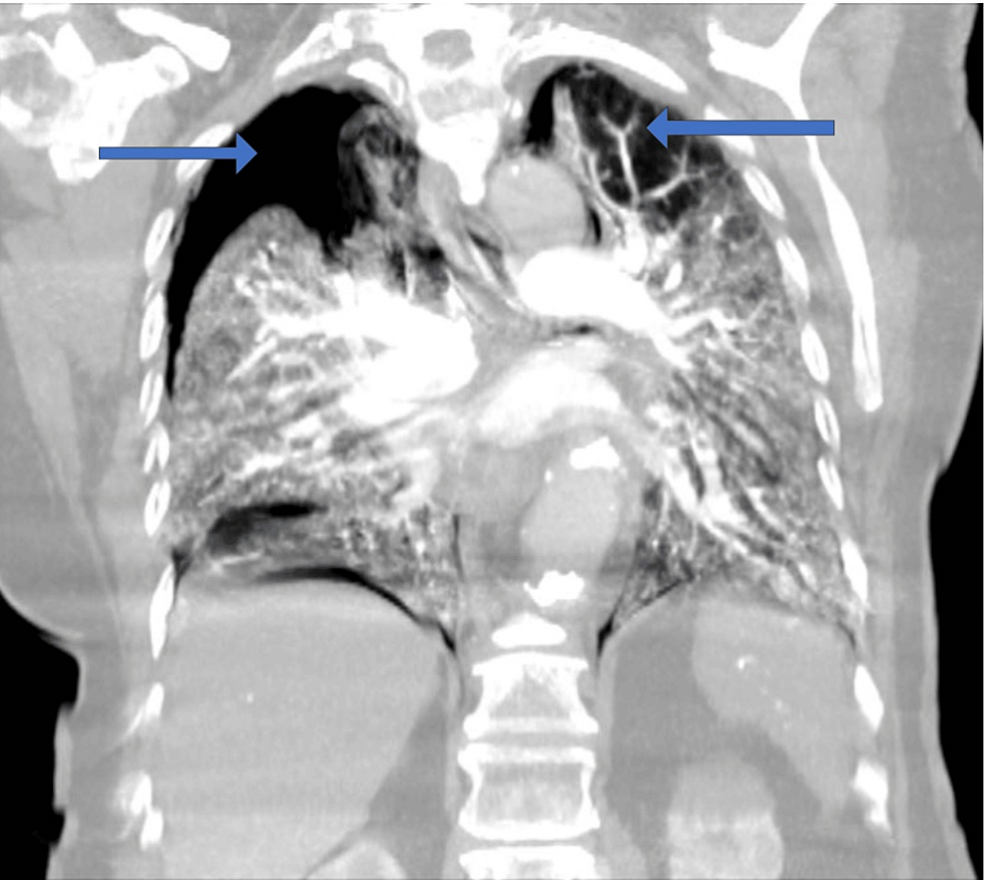
Repeat CT angiography of the chest showing bilateral pneumothorax with right greater than left.

Upon admission to the medical floor, pulmonology was consulted due to her continued oxygen requirements. Her oxygen saturation was 88% on 6 L/min via nasal cannula with normal blood pressure and heart rate. She tested positive for COVID-19, and due to worsening hypoxic respiratory failure, treatment with tocilizumab (because of elevated CRP), glucocorticoids, and diuretics was initiated. Since her symptoms had been present for longer than ten days, her timeline for antiviral treatment had elapsed; therefore, remdesivir was not indicated. Due to persistent hypoxia, she was started on a high-flow nasal cannula and moved to a long-term acute care facility for further management. On the 17th day of her hospitalization at our facility, the patient developed worsening hypoxic respiratory failure with a declining ROX index. Repeat pulmonary angiography was performed due to concerns for pulmonary embolism, which was negative, but it did reveal bilateral pneumothoraces (Figure 2). 

**Figure 3:**
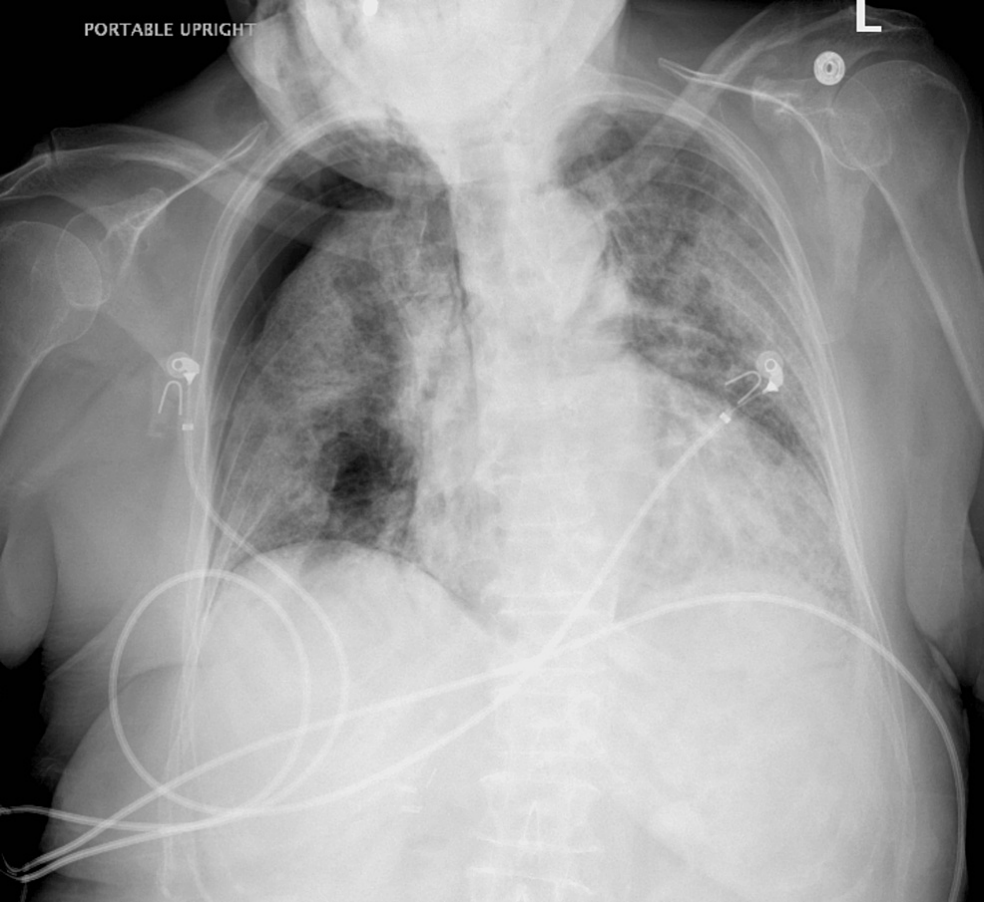
Chest radiograph showing a significant right pneumothorax.

She was monitored conservatively with observation, but due to the worsening of her condition, she was transferred to the intensive care unit (ICU) for emergent chest tube placement. Upon arrival at the ICU, her vitals were: SpO2 of 88%, respiratory rate of 35 breaths per minute, heart rate of 115 per minute, and temperature of 98°F. The complete hemogram was significant for leukocytosis of 18,000 with a neutrophil dominance at 87% and platelets of 160,000. An arterial blood gas (ABG) showed a compensated primary metabolic alkalosis with a pH of 7.48, PO2 of 67, PCO2 of 54, and HCO3 of 40 due to diuresis. Repeat imaging with a chest X-ray revealed a significant right pneumothorax (Figure 3). The chest tube was placed emergently in her right pleural space.

**Figure 4:**
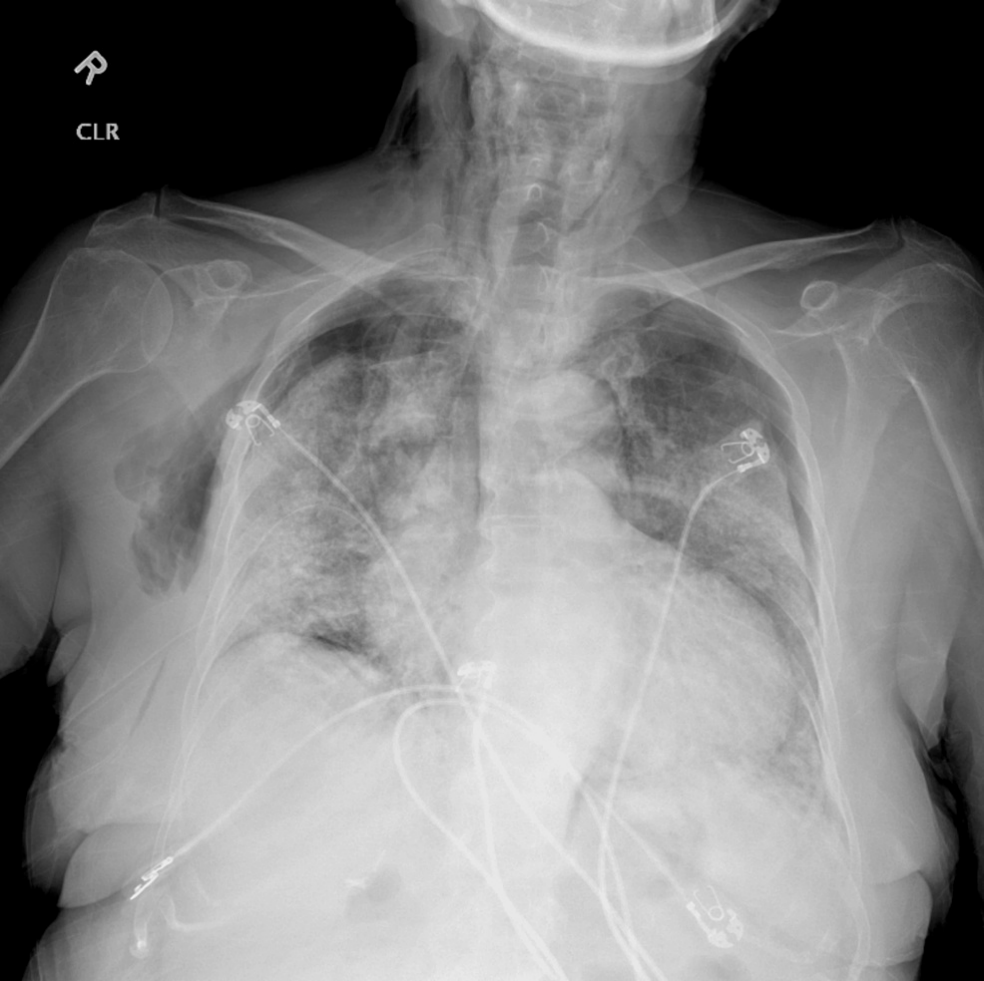
Chest radiograph showing bilateral pneumothorax, pneumomediastinum, and extensive subcutaneous emphysema in the neck and chest wall.

Her hospital course was further complicated by hypoxia and hypercapnia, requiring invasive mechanical ventilation. A repeat ABG obtained after intubation showed a new primary respiratory acidosis with a pH of 7.29, PCO2 of 81, PO2 of 114, and HCO3 of 39. A bedside chest X-ray revealed bilateral pneumothorax, pneumomediastinum, and extensive subcutaneous emphysema noted in the neck and chest wall (Figure 4). A second chest tube was placed in the left pleural space. Her FiO2 requirement was 100%, and PEEP was set at 14 cm H2O. The following day, the patient was placed on comfort measures due to poor prognosis and subsequently expired.

## Discussion

Spontaneous pneumothorax is defined as the presence of gas in the pleural space without an external traumatic or mechanical inciting event. It can be further divided into primary or secondary classifications. Primary spontaneous pneumothorax occurs without clinical lung disease, while secondary spontaneous pneumothorax usually occurs due to underlying lung disease. Both types of pneumothoraxes have a male preponderance; however, secondary spontaneous pneumothorax tends to occur in older patients [7]. Chronic obstructive pulmonary disease (COPD) is the most common cause of secondary spontaneous pneumothorax, with a reported 61% of cases according to a large epidemiological database study performed in England [7]. Other pulmonary infections causing secondary spontaneous pneumothorax include Staphylococcus, Klebsiella, Pseudomonas, Mycoplasma pneumoniae, and even Aspergillosis. Viruses such as influenza are also reported culprits. In HIV-infected patients or certain endemic populations, *Pneumocystis jirovecii *(PJP) and *Mycobacterium tuberculosis *can be potential causes as well. Interestingly, PCP infections can commonly result in bilateral pneumothorax. Less common causes of secondary spontaneous pneumothorax include diffuse cystic lung diseases resulting from lymphangioleiomyomatosis, Langerhans cell histiocytosis, Ehlers-Danlos syndrome, and neurofibromatosis [7-9]. Typical manifestations of pneumothorax include dyspnea, hypoxemia, pleuritic chest pain, and tachycardia [7,8]. 

The presenting patient had hypoxemia secondary to ARDS from COVID-19 infection complicated with bacterial pneumonia and pneumothorax. She had a medical history of hypertension, diastolic heart disease, diabetes mellitus type 2, chronic kidney disease stage 4, but otherwise no history of smoking or underlying pulmonary disease that could have contributed to her complications. The pathophysiological mechanism of COVID-19 resulting in spontaneous pneumothorax is complicated. The Macklin effect is the air dissection of the lung's bronchovascular tree from peripheral to central airways following injury to distal alveoli. Ultimately, the progression of the Macklin effect leads to the development of pneumomediastinum, subcutaneous emphysema, or pneumothorax. It has also been proposed to be related to diffuse alveolar damage resulting in alveolar rupture forming pulmonary blebs or bullae from an enormous inflammatory response, such as seen in a cytokine storm [8-10].

One of the most common causes of mortality with COVID-19 is a syndrome known as ARDS [2]. ARDS is a form of respiratory failure and non-cardiogenic pulmonary edema most commonly resulting from bacterial or viral pneumonia, but it can also arise from sepsis or trauma. Mortality related to ARDS is reported to be around 25-40% [11]. It has also been reported that patients with COVID-19 infection have had complications of spontaneous pneumothorax with both invasive and noninvasive ventilation [10,12]. This complication is usually a result of volutrauma and barotrauma in ARDS patients receiving invasive mechanical ventilation and has an incidence of about less than 1% in a study including 902 patients [10]. Regarding our patient, mechanical ventilation was necessary after she developed worsening hypoxic and hypercapnic respiratory failure, but this was late in the course, and the high volutrauma/barotrauma did not contribute to the development of bilateral pneumothorax typically seen in patients with high PEEP. The management of secondary spontaneous pneumothorax in COVID-19 has not been definitively elucidated yet but involves several different interventions, including oxygen support, tube thoracostomy, pleurodesis, or even invasive surgery, similar to the management of other types of pneumothorax [13].

## Conclusions

Bilateral spontaneous pneumothorax is a rare complication that can result from COVID-19 infection. This complication can potentially result from either invasive or noninvasive interventional forms of ventilation. It is important for physicians to be aware of this association to enable early recognition, diagnosis, and management.
